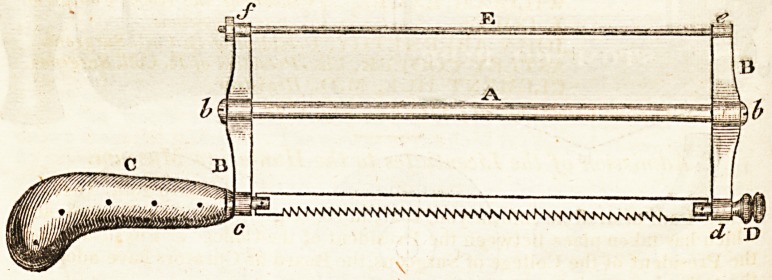# Intelligence

**Published:** 1827-04

**Authors:** 


### INTELLIGENCE.

### MONTHLY REPORT OF PREVALENT DISEASES.

In our last Report, we alluded to the great prevalence of pulmonary complaints: the acute attacks have become less frequent within the last fortnight, but many of the chronic affections continue their course without any disposition to yield. Chronic Bronchitis, especially in elderly persons, has been severe, and in several instances has given rise to dropsical effusions, of which, from this and other causes, we have met with many cases during the last two months. While the vicissitudes of the weather have produced these diseases in the aged or debilitated, those of more robust habit have been affected with Biliary Derangements and Diarrhœa, to a considerable extent: at least, were we to select any one set of complaints out of the common routine of winter cough and rheumatism, we would place these first on the list, as having been most prevalent during the period comprehended in our Report. Among the biliary cases, several have assumed the form of Jaundice; and one of these patients, the first of many to whom we have put the question, appears to see objects of a yellow tint. Most patients with jaundice will assert that white paper is yellow; because they think they ought to do so; but the young woman alluded to has invariably called every blue object green, which probably depends upon her seeing them through a yellow medium.

*March 26th.*

### *Reconciliation between* Mr. J. H. Green *and* Mr. Bransby Cooper.

As various papers relative to the unfortunate differences between these gentlemen have been laid before the public, we are happy in being able to inform our readers that a reconciliation has taken place, as will appear from the following document:—

"We, the undersigned, being anxious to reconcile the unfortunate misunderstanding existing between Mr. J. H. Green and Mr. Bransby B. Cooper, have, with the permission of these gentlemen, enquired into its origin, and have great pleasure in making the following communication to the profession.

"Upon a calm review of the occurrences which took place during the controversy respecting the election of an anatomical teacher at St. Thomas's Hospital, and in consequence of having learned that he had been misled by statements which he conceived at the time to be authentic, Mr. Bransby Cooper has expressed his conviction that Mr. Green's conduct was, throughout that controversy, guided by strict principles of honour and integrity, and has therefore acknowledged his regret that a misconception of Mr. Green's motives should have induced him to make any offensive animadversions on that gentleman's character.

"In consequence of this explanation on the part of Mr. Bransby B. Cooper, a mutual reconciliation has taken place, equally to the honour and satisfaction of both parties.

| | | |
|---|---|---|
| | B. C. BRODIE, | JOHN MORGAN, |
| (Signed) | BENJAMIN TRAVERS, | T. CALLAWAY. |
| | THOMAS ROSE, | |

*London; 13th March,* 1827."

---

### *Dissection of one of the Cases of Aneurism in which the Carotid Artery was supposed to have been tied beyond the Tumor.*

Our readers are probably aware that it was proposed by Dessault to tie the artery, in certain cases of aneurism, beyond the tumor, and that this operation was actually performed by Deschamps and by Sir A. Cooper; but, proving unsuccessful with them, never became generally adopted. Allusion is made in the present Number of the Journal to Mr. Wardrop's attempt to revive this method of operating; and we therefore think it right to make our readers acquainted with the state of parts, as discovered on the post-mortem examination of one of the recent cases.

The patient alluded to died last week, and the body was examined on the 23d, when it was found that *the carotid artery was pervious and undisturbed, presenting one continuous tube throughout, there being no unusual appearance, and no aneurism.* The heart was affected with Hypertrophy.

Mr. Travers, with reference to the alledged success of this method, remarks (page 331,) that it will be of much importance "*if borne out by similar results;*" and we have given the above details because it is obviously of great importance that surgeons should be able to form a true estimate of the value of any proposed method of treatment as soon as possible, that it may either be rejected or adopted, according to circumstances.

We are quite aware that mistakes will sometimes happen, even in the hands of skilful surgeons; and it is this consideration which has induced us to withhold *numerous other instances of unfortunate operations,* which have been transmitted to us for the purpose of publication, because they have not, like the present case, been connected with any important practical question.

*March 26th.*

---

### *Two Cases of Poisoning by Belladonna.* By Mr. Smith, Surgeon, of Forres, N. B.

Nov. 5 —At five P.M. I was called to see two of Mr. M.'s children, both boys, the one four, and the other two years of age. They had eaten, together with another child, of the berries of the Atropa Belladonna, from a bush in Mr. M.'s garden, to which they got access through a gap in the railing. It appears to have been between one and two o'clock that they were in the garden; for soon after two the elder M. went to school, where the symptoms to be detailed made their appearance. When taken up to his lesson, he did not speak, but laughed immoderately, and grasped at imaginary objects: he had, previous to this, complained of pain in his head. He was now sent home, where the laughing continued, and he was as talkative as he had before been silent, but he was altogether incoherent; added to this, he was in constant motion, running round and round the room. This wild conduct attracted the particular attention of his mother, who, observing a red stain on his face, suspected what had taken place. I found him laughing and talking alternately; he was now kept on the knee, but the extremities were in violent and almost constant action; the eyes fixed, and the pupil fully dilated, and insensible to the light of a candle. A scruple of Sulph. Zinc was immediately procured, and given at twice in the course of a few minutes; and, as soon as he began to vomit, the fauces and gullet were freely tickled with a feather. By these means a good deal of reddish matter, in which were many pieces of the berries, was brought up.

It was at this time that our attention (Mr. Adams, my partner, had arrived,) was called to the younger boy. In him the symptoms were the same, and now fully as violent. About half a scruple of Sulphate of Zinc was given to him, and the fauces and gullet treated in the same way. This caused him to vomit great quantities of porridge (which he had taken for dinner,) and one or two husks of the berries. To induce still more vomiting, a solution of Tart. Emetic was given to each. To effect this, and the giving of the other medicines, &c., as the jaws were firmly locked, it was necessary to separate them, and keep them so by the handle of a penknife. Besides the locking of the jaws, there was difficulty in swallowing, for but a very little milk was got down, although frequently administered. The titillation was continued at intervals, until both had evacuated a good deal of reddish looking matters; the colour being evidently caused by the juice of the berries. After some little time, when nothing more was coming up, about an ounce of Castor-oil was administered to each. Notwithstanding this treatment, the symptoms had in the mean time become worse. The muscular motions were stronger and incessant; breathing noisy, and with a croupy sound, and occasional cough; faces swollen and red; incoherent talking continuing. Soon after taking the oil, enemas were administered, and repeated about every two hours. They had also small quantities of vinegar and water (half and half,) given them frequently. It was now six o'clock. The elder boy's breathing was loud and stertorous, and the face much swollen; but the muscular motions were less violent and frequent; the skin cold; pulse barely perceptible from the beginning, now not felt in the radial artery: he was therefore put into a warm bath, and after a few minutes, while there, the jugular vein was opened, and some five or six ounces of blood taken away. This relieved him considerably. He was put into a blanket, and kept warm. There was now a disposition in both to sleep in the intervals of the muscular movements, which returned after short intervals of quiet; but it was not till towards the morning of the 6th, that we permitted them to take short sleeps. While not asleep, they were still incoherent. While awake, they had some strong coffee given them, or occasionally the vinegar and water.

6th.—About three o'clock this morning, more Castor-oil was ordered, but little got down. At nine, the elder boy had much croupy cough, which has caused, oftener than once, a little bleeding from the neck; locking of jaws less in both; other symptoms much the same. Coffee and enemas to be continued, and four grains of Calomel given to each. Shortly after this, the infant voided by stool about twenty skins of the berries, and, in the course of the forenoon, he had several feculent stools.

At two P.M. I found this poor little fellow cold, and deadly pale, with hardly any pulse. He was immediately put into a warm bath, and the chest rubbed with flour of mustard; an assafœtida enema was also thrown up. He gradually became warm, and the pulse more distinct. He was again in a state of collapse at six; when the same means were used, and he took small quantities of warm punch and chicken-broth. When taken from the water, he was wrapped in blankets, laid at the fireside, and bottles with hot water placed round him. At half-past seven, he was much revived, and asked for a drink; he also ate a spoonful or two of panado; preferred cold water for drink; still purged; stools watery. Some erithema, which was on him in the morning, has now disappeared.

At half-past four, the elder boy got another ounce of Castor-oil; he has been in a natural sleep for some time; has still slight convulsive motions; pulse very frequent, but distinct. Allowed plenty of tea, broth, or any liquid he likes; and to have a soap enema when he awakes.

7th.—Were both restless in the early part of the night, but have slept a good deal since morning; towards which, they began to distinguish objects, and to speak and act rationally. Previous to this they were blind, for the candle held close to the eye produced no effect on it, nor any appearance of their being aware of its presence. Pupils are still much dilated, and conjunctivæ red, although less so than they were. Pulses distinct, and in the eldest boy soft, and not very frequent. Freely purged, the infant complaining of some pain in the belly, which is not increased by pressure. Thirst great in both. The broth, &c. to be continued, and another enema administered to the elder.

From this time they continued to mend, and after a little time they had no complaint. The noisy, croupy cough continued longest; and, when the elder boy has a cold, the cough is still (at a distance of six years) of the same nature.

The boy mentioned as having partaken with the M.'s of the poison, was treated nearly in the same way by another practitioner, and with a like result.

*Forres, Feb.* 1827.

---

### *Case of Extra Uterine Fœtus.* *By* R. Mackie, Surgeon.

On the 9th October, 1826, a negress, about forty years of age, was brought into the Lying-in Hospital of Plantation, Richmond Hill, Leguan, to be delivered of her twelfth child. Next day, the midwife, thinking the labour rather tedious, sent for the medical practitioner of the estate, who, on examination per vaginam, concluded the patient not to be in labour. She complained of slight pain across the umbilical region, with numbness of the lower extremities. The vagina was not dilated beyond its natural size. Bowels empty, and urine passed freely; no constitutional irritation.

On the morning of the 10th, the patient was much in the same state as when admitted. The feeling of numbness in the lower extremities was changed to that of acute pain; the head of the child could be felt resting on the pubis, apparently covered by a thin membrane; the os tincæ could not be distinguished. Bowels regular; pulse natural.

In the evening, the symptoms continued much the same. No uterine pains; pulse still good. The patient was perfectly collected; partook of light food, and walked about the room, conversed cheerfully with her attendants, and was seemingly under no apprehension as to the result. It was thought necessary to administer a dose of Castor-oil, which operated freely about five o'clock. Soon after, in consequence of pain in the hypogastric region, a catheter was introduced into the bladder, and a small quantity of urine drawn off, which produced immediate relief. In introducing the catheter, it was found necessary to raise the head of the fœtus from the pubis.

During this time nothing had occurred to cause a supposition that any thing untoward was likely to take place. The case was considered as one of protracted labour; and, as the patient had had merely a few trivial pains, and was in full possession of her health, mental as well as bodily, it was hoped that, on the accession of true labour-pains, the child would be soon expelled.

At about seven in the evening of this day, while reclining on her back in a half-sitting posture, her extremities suddenly became cold, and, without making any complaint, or showing any symptom of being in pain, she, with one slight convulsion, expired.

On ascertaining the death of the mother, an incision was immediately made, about six inches in length, from the umbilicus downwards along the linea alba. On cutting through the peritoneum, a body presented in close contact therewith, which, on enlarging the opening, was protruded with considerable force, and proved to be a full-grown male child, with its funis and placenta. The child was perfect in every point, and was rather a large one. It was not enclosed in any sac, nor could any connexion be discovered between it and any part of the abdominal viscera. From the placenta being instantly expelled along with the fœtus, its attachment was not seen; nor did a subsequent examination lead to any discovery.

The uterus was found in its natural position, enlarged to about the size it attains in the latter end of the third month of pregnancy; the os tincæ dilated sufficiently to admit two fingers; its external surface presented no appearance of disease, nor was there the smallest sign of rupture ever having taken place in this viscus. The bladder was found empty; the placenta was flatter than usual. The position of the fœtus was with the breech to the navel of the mother, and the back extending along the linea alba; the head presenting at the superior aperture of the pelvis, pressing upon the anterior superior part of the vagina, and forcing backwards the os tincæ upon the sacrum. No further examination could be made. The uterus was extirpated at its connexion with the vagina, and is in the possession of the writer in the same state as when removed, together with the fœtus and placenta.

It should have been observed, that this woman, during pregnancy, suffered no sickness more than had been usual.

*Demerary; 12th Oct.* 1826.

---

### *An Account of an improved Stomach Pump, or Injecting Syringe, and of an Amputating Saw, invented by* Francis Fox, jun. M.D. House-Surgeon to the Derbyshire General Infirmary.

The instrument is constructed in the following manner:—The piston, with a square rod sliding through a square collar at the lid of the syringe, moves in a common cylinder, as in other syringes. The bottom of the syringe is composed of moderately thick brass, having two holes in it, through which the matter to be pumped passes: this circular brass end is turned on the outside at the end, and has another circular piece of brass, with two similar-sized holes in it; these two holes terminate in two projecting short pipes, to which the stomach tube and the basin tube are fixed. The touching surfaces of these two circular pieces of brass are ground together so as to fit air-tight, and to move smoothly on each other. To the lower or outer of these pieces, at its edge, is fixed a cylinder, which will fit on the outside of the barrel of the syringe without touching the same. This outer cylinder extends half way up the barrel of the syringe, and is grasped by the left hand when the instrument is in use, (this may be called the hand cylinder;) the right hand holds the handle of the piston-rod. The holes through the inner piece of brass are on each side the centre, but rather more than one diameter of one of the holes out of the line of the diameter of the brass; the two holes through the outer piece of brass are exactly in the line of its diameter, one on each side of its centre. The ground surface of the outer brass is kept in close contact with that of the inner brass, by a milled nut at the end; and there is a simple stop to prevent these two ground surfaces from revolving too far; so that when the stop acts in one direction, the hole on one side the centre is open, and when the stop acts in the other direction, the opposite hole is open, and so on alternately.

When the syringe is held in the proper position for use, that is nearly in the horizontal direction, the words STOMACH PIPE and BASIN PIPE are to be seen on the upper surface of the hand cylinder, and the word OPEN on the barrel of the syringe: thus, grasping the hand cylinder in the left hand, and the handle of the piston-rod in the right hand, by means of the square piston-rod the barrel of the syringe is turned at pleasure by the right hand, either one way or the other, so as to bring the words STOMACH PIPE in a line with the word OPEN, or the words BASIN PIPE and the word OPEN in a line with one another, indicating that the communication is open either with the stomach or with the basin; and only a very slight rotation of the right hand is required to effect this alternate change.

The two short tubes to which the stomach and the basin pipe are fixed, project straight out at the end of the syringe, having no angles or turns in them; the full bore of each is open alternately, and the other shut. When the milled nut is taken off the end, the hand cylinder, with the outer piece of brass, draws off the barrel, when both the ground surfaces are exposed to view, and can be cleaned in a moment if required, and as easily refixed for use.

The instrument is simple in construction, and has not any part which is liable to be out of order or to harbour dirt. The stomach and basin pipe are so fitted that the one cannot be put on to the place intended for the other, and the hand cylinder can only be put on in the proper way, in consequence of the stops; so that the pipes, and the words relating to them, are sure to be properly adjusted when the instrument is prepared for use. The syringe is perfectly uniform in its external appearance, being a straight barrel, with two tubes, half an inch long, projecting from its end; and may, in the strict sense of the word, be said to act without either valves or stop-cocks. The hands never require to be moved from their hold, and the course of the fluid acted upon is reversed at any moment, and with perfect ease, by a moderate turn of the right hand, and the words engraved on the syringe telling which pipe is open. Supposing it is intended to inject the stomach, the piston is to be drawn out, with the words BASIN PIPE and the word OPEN in a line; the syringe being full, the words STOMACH PIPE are to be opposite the word OPEN, and the piston forced into the barrel, when the fluid will pass into the stomach. The syringe can then be filled again from the basin, as just described, and so on. Or, by reversing the order of action,—viz. drawing out the piston when the words STOMACH PIPE, OPEN, are in a line,—the fluid will be drawn from the stomach. The course of the fluid is changed at any moment, by reversing the direction of the piston rod,—viz. by forcing it in or drawing it out. This will be found a sufficient description of the principle of construction, and method of using the instrument; and, by reference to the accompanying engraving, a further elucidation will be obtained.

In a paper, which was published in the London Medical and Physical Journal about two years ago, on the subject of the Stomach Pump, or Poison Syringe, I expressed the reasons why such instruments on the valve principle were all to be condemned: first, because the direction of the fluid acted upon cannot be reversed without removing and changing the position of the pipes, which reverse is continually necessary in extracting the contents of the stomach, in consequence of the end of the stomach-pipe becoming often stopped up, by the lumpy and fibrous matter which the stomach so constantly contains. Secondly, because the valves must in a degree obstruct the passage of a thick, lumpy, and fibrous pulp, and even be occasionally propped open by the same. And, thirdly, because valves may be out of order, and rectifying them requires considerable attention. But I repeat, by far the most important and insurmountable objection to valves is, that the course of the fluid cannot be alternated with expedition, which every practical man must know to be absolutely essential during the extraction of the contents of the stomach. I stated in the paper here alluded to, that valve syringes were elegant instruments, where the passage of clear fluids only was required. Under this conviction, founded upon experience in the use of the stomach pump, I recommended one with a double stop-cock, moved by the forefinger of the left hand, whilst grasping the syringe, so that the pipes were alternately opened and shut by each movement of the finger. This instrument has been publicly noticed by a writer, who stated *that valves were the only things to be recommended in the construction of a stomach pump*. These assertions, however, are not to be taken on the authority of the maker of the instrument, but experience and observation alone must decide.

Syringes on the stop-cock plan afford free passage to thick and fibrous pulp; the course of the fluid is instantly changed by reversing the direction of the piston; and, had the mode of alternately opening the cocks been sufficiently easy and simple to the operator, at the same time there being no complication in construction, the instrument would have been perfect: but, as I have not seen all these points satisfactorily accomplished, I have turned my attention to the subject, and take this opportunity of submitting my improved syringe to the opinion of the medical profession, and only request that it shall have a fair and impartial trial and examination, knowing that its fate will then depend upon its merits or demerits. This syringe, from the freeness of its passages, and from there being no part in it which can harbour dirt, is recommended for the injection of large anatomical subjects; as a syringefull of injection could be repeatedly forced in without removing it from the vessel tube, and hence all breaks in the injection would be avoided, and the operation expedited. I shall now only add, that the instrument is applicable for all purposes where rather a large syringe is convenient.

The syringe is recommended to be eight inches and a half long, and the barrel two inches in diameter; the other parts larger than the drawing, in the same proportion.

*Description of the Engraving.*—A, The milled nut which keeps the hand cylinder B in its situation on the barrel of the syringe.

C, C, The two rockets, with a portion of the elastic stomach pipe attached to one, and a portion of the basin pipe affixed to the other: these rockets fit on to the projecting tubes D, D.

E, F, represent the surfaces which are ground together of the two circular pieces of brass at the bottom of the syringe, in which the position of the holes is displayed. The centre circle in E represents the pivot which moves in the hole shown in the centre of F.

G, H, denote the groove and pin forming the stop. These two circular pieces of brass are represented as detached from the ends of the cylinders, to simplify the engraving.

### *Description of the Saw.*

The principle of this saw is the same as the one used by joiners and cabinet makers for sawing circular and curved pieces of wood, where the end bars and the middle rod are made of wood, and the saw-blade is stretched by twisting a double string, by a small wooden lever placed between the two strings. It is not necessary to describe this principle more accurately, as any one, by reference to the joiner's saw in question, will understand it immediately.

The objects aimed at in the construction of this amputating saw, have been to render the instrument as light as possible, and at the same time to ensure the powerful extension of the saw-blade; and also that the stretching part of the saw shall admit of turning to any desired angle with the plane of the blade, so that the saw-blade may be applied to the bone, *quite close up to the muscles,* without the fleshy parts held back by the retractors coming in contact with the stretching part (or back of the saw). This circumstance renders the saw particularly convenient in amputations with the flap; as, in muscular subjects, the flap is often found to be in the way of the back parts of the broad-bladed saw in common use.

The objection to the bow amputating saw, is that the bow requires to be so thick to secure the powerful stretching of the blade, that the weight of the instrument is very considerable, and this weight, when entirely rested on the bone, frequently causes the teeth to take so much hold as to fasten the saw during the operation. The bow amputating saw at this Infirmary weighs one pound; the one on the construction here described, weighs only half a pound.

These observations, and the amputating saw here recommended to the consideration of the profession, are the result of the frequent opportunities which I have had, in my official capacity, of witnessing amputations of various descriptions. The instrument is introduced before the public with all due deference to the opinions of the members of the profession, with a full conviction that these suggestions will lead to improvement, although the saw here recommended may be thought to require some modifications in construction.

*Observations on the Engraving, (which represents the relative size of every part.)*—A, Is an iron tube, to render it light, and to ensure its being strong for its weight.

B, B, are two steel plates, or bars, into which the tube A fits, and is secured by two screws at *b, b.*

C, the wooden or ivory handle of the saw, with a steel shank, which passes through, and turns very tight in the end of the steel plate at *c.*

D, a milled double nut, which turns very tight in the end of the plate at *d.* This double nut is constructed so as to admit of the accurate adjustment of the length of the saw-blade, that the two steel plates B, B, shall be square with the tube A, when the blade is on the *full stretch.* This will be easily comprehended by examining an instrument constructed according to this principle.—The saw-blade is pinned into a slit in the handle-shank and into the nut-shank, so as to admit of being turned to any desired angle, by means of the handle and double-milled nut.

E, The stretching rod, made of very thin steel wire, is fast in the plate at *e,* passes through a hole at *f,* and has a small nut tapped on to its end; which nut is screwed up by a small wrench, or key, so as to put the blade on *full stretch.*

The thinner the blade is, the better.

### *Annual Report of the National Vaccine Board.*

To the Right Honourable Robert Peel, Secretary of State for the Home Department.

Sir,—We continue to use all possible diligence in extending the knowledge of the best process for effectual vaccination, and to supply the means, as well as to suggest the mode, of accomplishing this object.

From the quantity of vaccine lymph distributed since our last Report, and from the accounts of our correspondents, we are led to presume that this practice is becoming daily more general; and this inference is still further confirmed by the fact, that, within the last twelve months, only 503 deaths have occurred from small-pox within the Bills of Mortality; whereas, in the preceding year, 1299 persons are recorded as having fallen victims to that loathsome disease. The whole of this difference ought not, perhaps, in candour, to be attributed to the influence of vaccination; for the small-pox, during the year 1825, assumed a peculiarly malignant character; and there were more instances of that distemper occurring *twice* in the same individual, than had ever been reported to us before. But when we reflect that, before the introduction of vaccination, the average number of deaths from small-pox, within the Bills of Mortality, was annually about 4000, no stronger argument can reasonably be demanded in favour of the value of this important discovery. Nor can any more striking proof be given of the paternal care of government to protect the people at home and abroad from this destructive disease, than the establishment and maintenance of this Board.

We have the honour to be, Sir, your faithful servants,

HENRY HALFORD, *President of R. Coll. Physicians.*
WILLIAM LAMBE, } *Censors of the Royal College of*
J. COPE, } *Physicians.*
JOHN ABERNETHY, *President of R. Coll. Surgeons.*
ASTLEY COOPER, *Vice-President of R. Coll. Surgeons.*
CLEMENT HUE, M.D. *Registrar.*

*National Vaccine Establishment;* 17*th February,* 1827.

---

### *Admission of the Licentiates to the Hunterian Museum.*

*February* 20*th,* 1827.

Sir,—I am desired to inform you, that, in consequence of a correspondence which has taken place between the President of the College of Physicians and the President of the College of Surgeons, the Board of Curators have adopted the following resolution:

"That the Licentiates of the College of Physicians shall hereafter be admitted to the Museum of the College of Surgeons, upon all days of public exhibition, without further ceremony than that of inscribing their names in the visitors' book."

I am, Sir, your humble servant,
WM. MACMICHAEL, *Registrar.*

*By order of the Royal College of Physicians.*

### METEOROLOGICAL JOURNAL,

*From February 20th, to March 20th,* 1827.

By Messrs. HARRIS and Co. Mathematical Instrument Makers, 50, High Holborn.

| February | Rain gauge. | Moon. | Thermom. | | | Barometer. | | De Luc's Hygrom. | | Winds. | | Atmospheric Variations. | | |
|---|---|---|---|---|---|---|---|---|---|---|---|---|---|---|
| | | | 9 A.M. | MAX. | MIN. | 9 A.M. | 10P.M. | 9 A.M. | 10P.M. | 9 A.M. | 10PM. | 9 *a.m.* | 2 *p.m.* | 10 *p.m.* |
| 20 | | | 28 | 32 | 32 | 29.56 | 29.54 | 82 | 80 | ENE | NE | Fair | Fair | Cloudy |
| 21 | | | 34 | 38 | 32 | 29.53 | 29.61 | 89 | 87 | NE | ENE | Cloudy | — | Fair |
| 22 | | | 35 | 39 | 25 | 29.75 | 29.87 | 84 | 78 | N | NNW | Fair | — | Cloudy |
| 23 | | | 32 | 38 | 29 | 29.93 | 29.89 | 74 | 72 | W | SW | — | — | Foggy |
| 24 | | | 35 | 40 | 26 | 29.84 | 29.92 | 75 | 76 | NE | ESE | Foggy | — | — |
| 25 | | ● | 34 | 40 | 34 | 30.04 | 29.95 | 79 | 77 | SE | SSE | — | Fine | — |
| 26 | | | 38 | 50 | 47 | 29.73 | 29.65 | 92 | 85 | S | WSW | Rain | Fair | Cloudy |
| 27 | | | 50 | 54 | 36 | 29.39 | 29.57 | 94 | 92 | WSW | W | Cloudy | Rain | — |
| 28 | | | 37 | 49 | 49 | 29.56 | 29.31 | 98 | 98 | ESE | SW | Rain | — | Rain |
| Mar. | | | | | | | | | | | | | | |
| 1 | | | 50 | 44 | 44 | 29.23 | 29.34 | 82 | 97 | SW v. | SW | Cloudy | — | — |
| 2 | .16 | | 47 | 48 | 39 | 29.17 | 29.42 | 97 | 87 | SSW | SSW | Rain | — | Fair |
| 3 | | | 45 | 48 | 43 | 29.35 | 29.03 | 90 | 92 | SW | ESE | Cloudy | Cloudy | Cloudy |
| 4 | | | 46 | 49 | 33 | 28.71 | 29.20 | 93 | 83 | SSW | SW v. | — | — | — |
| 5 | | ☽ | 35 | 45 | 44 | 29.62 | 29.21 | 83 | 92 | WSW | SW v. | Fair | — | — |
| 6 | .24 | | 49 | 51 | 39 | 28 97 | 28.98 | 91 | 81 | SW | N to S | Rain | Rain | Rain |
| 7 | | | 40 | 51 | 43 | 29.43 | 28.94 | 87 | 95 | SW | S | Overca. | Cloudy | — |
| 8 | | | 46 | 51 | 34 | 28.79 | 29.10 | 84 | 78 | SW | WNW | — | — | — |
| 9 | | | 37 | 41 | 32 | 29.30 | 29.35 | 80 | 83 | NW | ENE | Fair | Fair | Cloudy |
| 10 | | | 41 | 45 | 37 | 29.66 | 29.69 | 78 | 78 | ESE | SSE | — | — | — |
| 11 | | | 50 | 56 | 47 | 29.44 | 29.40 | 87 | 95 | SW | SW v. | Cloudy | — | — |
| 12 | | | 49 | 55 | 46 | 29.56 | 29.81 | 88 | 87 | W | SW | Fair | Fine | — |
| 13 | .8 | ○ | 50 | 56 | 44 | 29.74 | 29.62 | 88 | 95 | NW | WSW | — | Fair | Rain |
| 14 | | | 48 | 51 | 43 | 29.65 | 29.84 | 77 | 82 | NW | W | — | — | Cloudy |
| 15 | | | 45 | 48 | 35 | 29.50 | 29.71 | 87 | 78 | W | WNW | Sm. Ra. | — | Fine |
| 16 | | | 40 | 48 | 43 | 30.01 | 29.76 | 80 | 92 | W | SW | Fair | — | Rain |
| 17 | | | 47 | 47 | 34 | 29.25 | 29.65 | 85 | 83 | WNW | WNW | — | — | Fair |
| 18 | | | 40 | 44 | 32 | 29.92 | 30.12 | 75 | 77 | NW | NNE | — | — | — |
| 19 | | | 40 | 45 | 43 | 30.20 | 30.19 | 77 | 84 | W | SW | — | — | Cloudy |

## Figures and Tables

**Figure f1:**
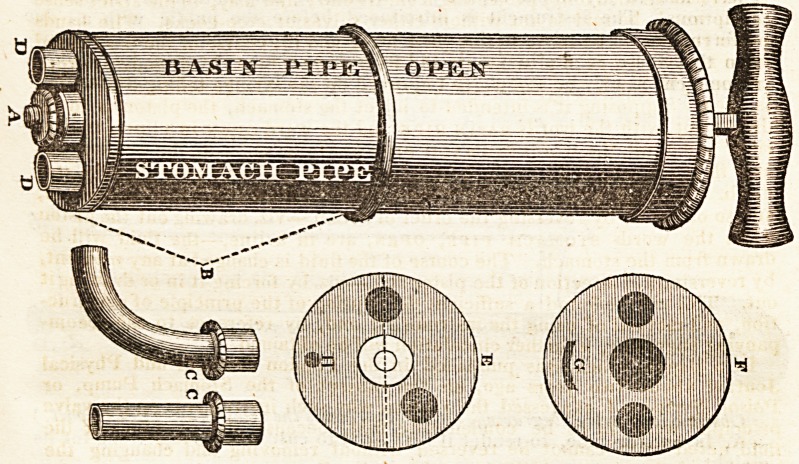


**Figure f2:**